# Temporal and tissue-specific variability of SMN protein levels in mouse models of spinal muscular atrophy

**DOI:** 10.1093/hmg/ddy195

**Published:** 2018-05-22

**Authors:** Ewout J N Groen, Elena Perenthaler, Natalie L Courtney, Crispin Y Jordan, Hannah K Shorrock, Dinja van der Hoorn, Yu-Ting Huang, Lyndsay M Murray, Gabriella Viero, Thomas H Gillingwater

**Affiliations:** 1 Centre for Discovery Brain Sciences, Edinburgh Medical School: Biomedical Sciences; 2 Euan MacDonald Centre for Motor Neurone Disease Research, University of Edinburgh, Edinburgh EH8 9XD, UK; 3 Institute of Biophysics, CNR Unit at Trento, 38123 Povo, Trento, Italy

## Abstract

Spinal muscular atrophy (SMA) is a progressive motor neuron disease caused by deleterious variants in *SMN1* that lead to a marked decrease in survival motor neuron (SMN) protein expression. Humans have a second *SMN* gene (*SMN2*) that is almost identical to *SMN1*. However, due to alternative splicing the majority of *SMN2* messenger ribonucleic acid (mRNA) is translated into a truncated, unstable protein that is quickly degraded. Because the presence of *SMN2* provides a unique opportunity for therapy development in SMA patients, the mechanisms that regulate *SMN2* splicing and mRNA expression have been elucidated in great detail. In contrast, how much SMN protein is produced at different developmental time points and in different tissues remains under-characterized. In this study, we addressed this issue by determining SMN protein expression levels at three developmental time points across six different mouse tissues and in two distinct mouse models of SMA (‘severe’ *Taiwanese* and ‘intermediate’ *Smn^2B/−^* mice). We found that, in healthy control mice, SMN protein expression was significantly influenced by both age and tissue type. When comparing mouse models of SMA, we found that, despite being transcribed from genetically different alleles, control SMN levels were relatively similar. In contrast, the degree of SMN depletion between tissues in SMA varied substantially over time and between the two models. These findings offer an explanation for the differential vulnerability of tissues and organs observed in SMA and further our understanding of the systemic and temporal requirements for SMN with direct relevance for developing effective therapies for SMA.

## Introduction

Spinal muscular atrophy (SMA) is a progressive motor neuron disease (1). The most common form of SMA (∼50% of all cases; type I) occurs in infants and is characterized by disease onset before the age of 6 months and, when untreated, leads to death before the age of 2 years. SMA is caused by the homozygous deletion of *SMN1* (∼95%) or other mutations in *SMN1* (∼5%) that lead to a loss of survival motor neuron (SMN) protein expression (2). Humans have a second *SMN* gene (*SMN2*) that is almost identical to *SMN1*. However, due to a C-to-T transition at the 5′ end of exon 7, exon 7 is removed by splicing from the majority of *SMN2* transcripts (3,4). The truncated *SMN2* messenger ribonucleic acid (mRNA) product is translated into an unstable protein that is quickly degraded (5). In contrast, a minority (∼10%) of *SMN2*-derived mRNA includes exon 7, which is subsequently translated into full-length, stable SMN protein. The number of *SMN2* copies in the human genome is variable, and therefore, the number of *SMN2* copies is the main determinant of the SMA phenotype: a higher number of *SMN2* copies correlates with higher levels of full-length SMN which, in turn, correlates well with the observed clinical phenotype (6). As such, the presence of *SMN2* provides a unique opportunity for therapy development and the mechanisms that regulate *SMN2* splicing have been studied in great detail (7). Excitingly, this led to the recent approval of nusinersen (Spinraza) for the treatment of SMA (8). Nusinersen is an intrathecally delivered antisense oligonucleotide that targets an intronic element upstream of exon 7, leading to increased inclusion of exon 7 and increased levels of full-length SMN protein. This is a milestone development that leads to meaningful clinical improvement in a large group of SMA patients (9,10).

In contrast, how SMN protein is expressed at different developmental time points and in different tissues is still poorly understood but remains important for several reasons. First, although SMA is primarily a motor neuron disease, other cell types and tissues are affected to varying degrees (11). The extent to which this is related to varying SMN requirements between tissues remains unknown; in general, RNA levels do not always linearly correlate with protein levels and the amount of protein translated from RNA varies between tissues (12). Second, a better understanding of the relative SMN requirements in different tissues will help guide the targeting of SMA therapy delivery: for example, nusinersen is currently delivered directly to the central nervous system (CNS) but this may not be sufficient to achieve optimal therapeutic efficacy (13). Finally, recent research has identified many cellular pathways that are affected by SMN depletion in SMA (14,15). Although these findings contribute to advancing our understanding of SMA pathogenesis, they also illustrate the increasing need to identify common pathways leading to pathology in SMA. Generating a reference dataset of temporal and tissue-specific SMN expression levels will help to interpret and prioritize the ongoing functional research into the downstream cellular consequences of SMN depletion.

Previous work has found that SMN protein levels are, as expected, significantly reduced in tissue from SMA patients and across disease models (Table 1). However, these studies are challenging to compare directly, as: (1) different techniques have been used to determine SMN levels, (2) when western blot was used to determine SMN levels, varying loading controls were used, (3) the number of studies that determine SMN levels at different time points is limited and (4) often SMN expression was determined in limited numbers of samples or replicates that makes results difficult to interpret quantitatively. In this study, we aimed to systematically address these issues by determining SMN protein expression in six different tissues and at three time points in both healthy control mice, and in two distinct mouse models of SMA. We used these results to first establish SMN protein levels in control mice and subsequently quantify the relative decrease in SMN expression at each of the time points in each of the investigated tissues for both models. Our findings suggest that better understanding and appreciating the significant variability in SMN expression in tissues, over time and between models can provide a way to better understanding the differential vulnerability of tissues and cellular pathways to SMN depletion.

**Table 1.** Overview of previous studies investigating SMN protein levels

Study	Tissue	SMA type	*n* patients	SMN level % of control	Method	Comment
Lefebvre *et al.* (35)	Liver	I (fetal tissue)	2	7.5–12.5%	WB—SMN/beta-tubulin	
		III (fetal tissue)	1	37.5%		
	Spinal cord	I (fetal tissue)	2	22–23%		
		III (fetal tissue)	1	39%		
Coovert *et al.* (34)	Spinal cord	I	4	0.9–4%		
	Muscle	I	4	reduced	WB—SMN/beta-tubulin	
		III	1	unchanged		
Burlet *et al.* (33)	Various tissues	Control (fetal tissue)	1	–	WB—SMN/beta-tubulin—actin	SMN is variable in different tissues
	Muscle	I	3	∼0%	WB—SMN/beta-tubulin	
		II	3	∼15–20%		
		III	2	∼15–20%		
Mutsaers *et al.* (36)	Muscle (quadriceps femoris)	II/III	3	∼30%	WB—SMN/beta-V-tubulin	
Ebert *et al.* (43)	iPSC-derived motor neuron	I	1	∼23%	WB—SMN/actin	
Liu *et al.* (44)	iPSC-derived motor neuron	I	3	∼20–25%	WB—SMN/NSE (neuronal marker)	
Coovert *et al.* (34)	Fibroblasts	I	5	9–27%	WB—SMN/beta-tubulin	
		II	8	15–41%		
		III	1	63%		
Crawford *et al.* (41)	PBMC	Type I–III	105	∼50–70% (estimate)	ELISA	
Also-Rallo *et al.* (40)	Fibroblasts	Type I/II/III/IV	1/1/4/1	variable	WB—SMN/GAPDH	40–50% SMN of controls in type I/II, variable/unchanged in other types
	Lymphoblasts	Type I/II/III/IV	1/1/4/1	variable		
Kobayashi *et al.* (37)	PBMC	Type I	4	∼12%	ELISA	
Zaworski *et al.* (38)	Whole blood	Type I/II/III	5/22/22	∼40%	SMN-ECL	
Wadman *et al.* (42)	PBMC	Type I/II/III/IV	18/60/52/5	69%	ELISA	
	Fibroblasts	Type I/II/III	5/19/16	66%	ELISA	

Study	Tissue	SMA model	Time points	SMN level % of control	Method	Comment

Kobayashi *et al.* (37)	Brain	delta7	P3—P9—P14	43—25—11%	ELISA	Decrease over time in all delta7 tissues
	Spinal cord		P3—P9—P14	38—34—28%		
	Muscle (gastrocnemius)		P3—P9—P14	19—8%—N/A		SMN expression undetectable at P14
Zaworski *et al.* (38)	Brain	C/C	P3–P120	unchanged–∼50%	SMN-ECL	Decrease over time in all tissues investigated
	Spinal cord		P14–P120	∼17–30%		
	Muscle (gastrocnemius)		P14–P120	comparably low between WT and SMA		
	Whole blood		P3–P120	∼50–25%		
Bowerman *et al.* (28)	Brain	*Smn2B/−*		9%	WB—SMN/actin	
	Spinal cord			24.5%		
Mutsaers *et al.* (36)	Muscle (gastrocnemius)	Taiwanese	P10	∼25%	WB—SMN/beta-V-tubulin	
Eshraghi *et al.* (31)	Spinal cord	*Smn2B/−* (FVB/BL6)	P5–P9	∼10%	WB—SMN/GAPDH	

Previous studies were identified in which SMN levels were determined in patient tissue, cell lines and/or SMA animal models. Studies were included when protein levels had been investigated using methods and sample numbers that allow for SMN protein levels to be quantified or estimated. The ‘method’ column includes loading controls used when SMN levels were determined via western blot (WB).

## Results

### Quantification of protein levels across tissues and throughout development

**Figure 1.**
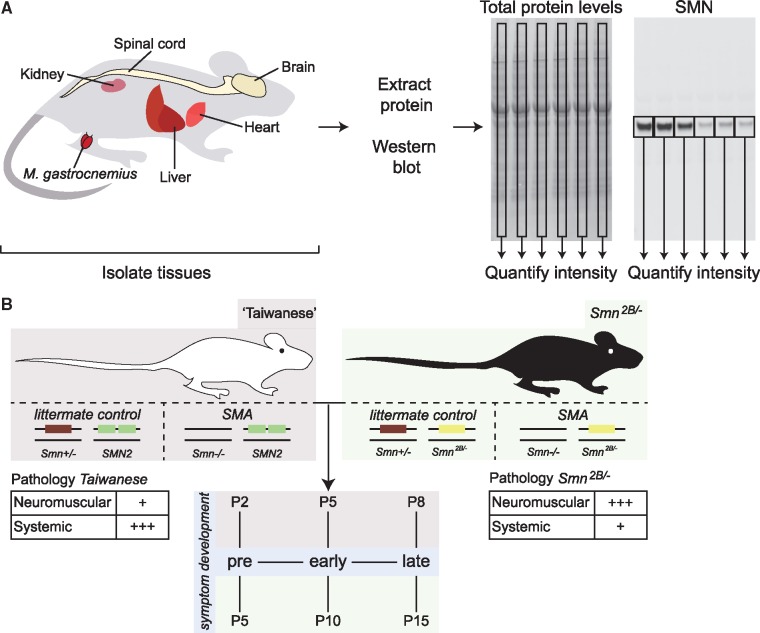
** **Methods and mouse models used to determine SMN protein levels. (**A**) Overview of tissues collected from both mouse models used in this study. Tissues to be analysed by quantitative western blotting included brain, spinal cord, skeletal muscle (*M. gastrocnemius*), heart, liver and kidney. (**B**) *Taiwanese* and *Smn^2B/−^* models of SMA were used in this study. The *Taiwanese* model is based on a human transgene (*SMN2*), whereas the *Smn^2B/−^* model is based on a mutant mouse *Smn* allele. Phenotypically, both models reflect core features of SMA, however, the *Taiwanese* shows a more pronounced systemic phenotype whereas the *Smn^2B/−^* model shows a clearer neuromuscular phenotype (32).

Accurate quantitative assessment of protein levels by western blot requires a carefully controlled, reproducible and reliable normalization method to account for differences in transfer efficiency and gel loading. Traditionally, housekeeping proteins such as actin, tubulin and glyceraldehyde-3-phosphate dehydrogenase (GAPDH) have been used to normalize protein levels for quantification purposes but these have several disadvantages. First, the expression of housekeeping genes has been shown to vary considerably between tissues, which makes it difficult to select a single loading control for use across a range of tissues (16). Moreover, housekeeping proteins show varying expression levels during development, which complicates comparisons of protein levels obtained at different developmental time points (17). Finally, many household proteins have been shown to change in disease conditions. Indeed, several commonly used housekeeping proteins (actin, tubulin and GAPDH) have been shown to be decreased in cellular models of SMA and in tissues from SMA mouse models (16,18,19). To reliably normalize protein levels, total protein stains (TPSs) (such as coomassie, ponceau S or fluorescently detectable variants) that allow quantification of the total protein loaded are therefore preferable (20). As in this study we aimed to quantify protein levels across tissues and at different postnatal developmental time points, we made use of a sensitive, quantitative western blotting method based on laser-scanning detection of fluorescently labelled secondary antibodies and a fluorescent TPS. This allowed us to normalize and quantify SMN levels across tissues and throughout postnatal development (Fig. 1A). Fluorescent TPSs and uncropped SMN western blots for all SMN blots in each of the figures are included in Supplementary Material, Figures S1–S5.

### Variation in SMN expression between CNS and peripheral tissues in control mice

First, we investigated SMN protein levels in control conditions by using control littermates from the *Taiwanese* mouse model of SMA (21). In this model, control and SMA-like mice are obtained by breeding mice that are heterozygous for mouse *Smn* (*Smn+/−*) with *Smn*-null mice that carry two alleles with two copies of *SMN2* (*Smn−/−; SMN2^tg/tg^*, total 4 copies *SMN2*) on a congenic FVB background (22,23). Experimental litters subsequently consist of ∼50% control littermates (*Smn+/−; SMN2^tg/0^*, total one allele mouse *Smn* and two copies of *SMN2*) and ∼50% severe SMA-like littermates (*Smn−/−; SMN2^tg/0^*, no mouse *Smn* and two copies of *SMN2*). Control littermates develop completely normally (21). Severe SMA-like littermates are initially not distinguishable from controls. However, after several days a severe neuromuscular and systemic phenotype develops, characterized by progressive hind limb paralysis and extensive organ pathology (Fig. 1B, left panel). The timing of the disease process varies slightly between colonies, however, in our colony mice start developing symptoms around postnatal day 4 (P4), which progressively worsen and lead to an average survival of 8–9 days postnatally (P8/P9) (24). For this study, we therefore defined pre-symptomatic as P2, early symptomatic as P5 and late symptomatic as P8.

**Figure 2.**
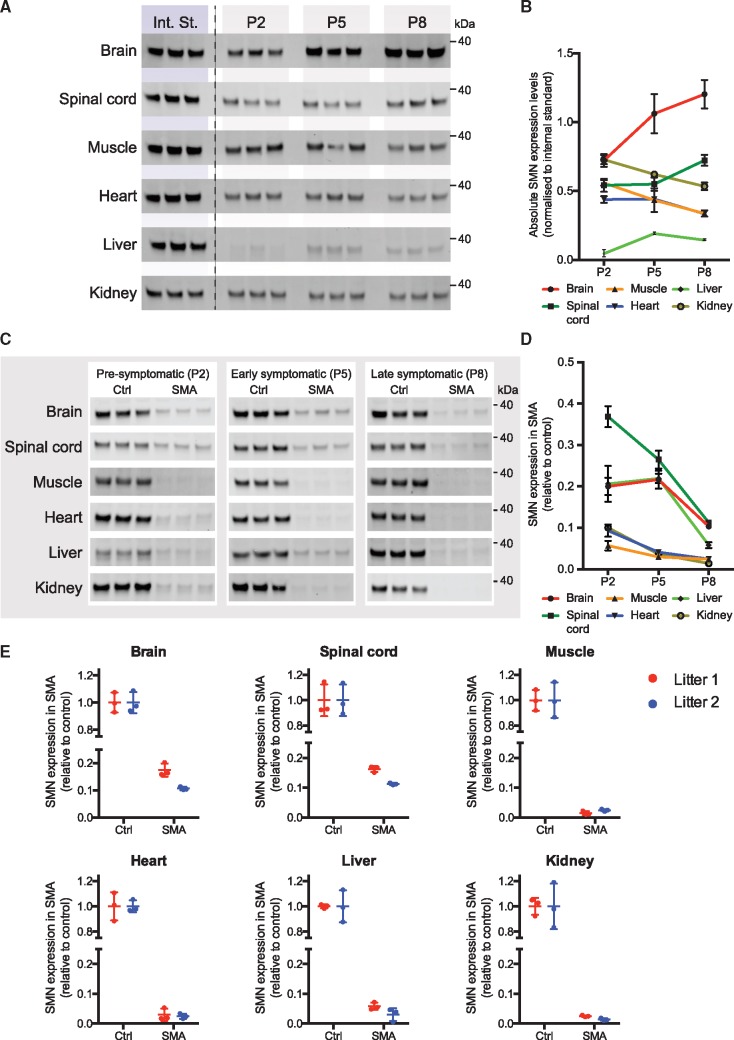
Analysis of SMN expression in control and Taiwanese SMA mice. (**A**) SMN expression in control littermates (*Smn+/−; SMN2^tg/0^*) of the *Taiwanese* model of SMA was determined using western blot in the indicated tissues. Shown are the internal standard (Int. St.) that was the same for all tissues and loaded in triplicate on each membrane, and SMN expression for each of the tissues at each of the indicated time points (P2, P5 and P8). (**B**) Quantification of SMN expression in all tissues from (A). Expression was first normalized to a fluorescent TPS and then to the Int. St., to allow direct comparison between membranes (*n* = 3 biological replicates for each of the tissues at each of the indicated time points, ± SEM). All tissues for each time point were obtained from the same litter. (**C**) SMN expression is shown for each of the indicated tissues at pre-, early and late-symptomatic ages (P2, P5 and P8) for both control (*n* = 3 for each tissue and each time point, *Smn+/−; SMN2^tg/0^*) and SMA (*n* = 3 for each tissue and each time point, *Smn−/−; SMN2^tg/0^*) mice. (**D**) Quantification of relative SMN depletion in each of the tissues at each of the time points. SMN expression in each sample was first normalized to a fluorescent TPS. SMN expression in SMA littermates is shown relative to control. (**E**) SMN levels were determined in each of the six tissues in two independent litters at the late-symptomatic time point (P8). kDa: 40 kDa molecular weight marker, see Supplementary Material for uncropped western blot membranes.

To establish between-tissue variability of SMN expression, we determined SMN protein levels in three biological replicates from control littermates (*Smn+/−; SMN2^tg/0^*) at the disease-relevant time points P2, P5 and P8. We investigated SMN expression in six different tissues: brain, spinal cord, muscle (*M. gastrocnemius*), heart, liver and kidney (Fig. 2A and Supplementary Material, Fig. S1). In addition to normalizing SMN levels to TPS, samples were normalized to an internal standard to account for variability between membranes. The internal standard used in this and in subsequent experiments consisted of a mixture of P5 brain lysates (see Materials and Methods for further details) and allowed us to quantify and directly compare SMN expression in each of the investigated tissues (Fig. 2B). We found that the effect of age on SMN expression significantly depended on the tissue investigated and, vice versa, the effect of tissue on SMN expression significantly depended upon the age investigated (*P* < 0.001). To examine this effect further, we performed *post hoc* analyses to determine individual *P*-values for each of the time and tissue comparisons (Supplementary Material, Table S1). At P2, SMN levels were relatively similar in all of the tissues investigated, except in liver where SMN levels were significantly lower. However, at later time points substantially different patterns in SMN expression occurred. Brain and spinal cord SMN levels increased significantly from P2 to P8. In contrast, SMN levels in muscle and kidney decreased from P2 to P8 whereas heart SMN levels remained constant. SMN expression in liver was significantly lower at all times. These results indicate that SMN requirements are likely different between nervous tissue and other types of tissues over the normal process of postnatal development and maturation in mice (*Mus musculus*).

### *SMN depletion varies between tissues but is consistent across litters in* Taiwanese *SMA mice*

After observing a surprisingly large variability in SMN expression across tissues in control mice, we wanted to establish whether similar variation was present in SMA mice. To assess this, we determined the amount of SMN in SMA mice relative to control littermates in each of the investigated tissues at P2, P5 and P8 (Fig. 2C andSupplementary Material, Fig. S2). SMN levels were quantified in three SMA mice and three controls by first normalizing expression to fluorescent TPS and then normalizing SMN levels in SMA to the average of three control littermates (Fig. 2D, for percentages see Fig. 4C). Again, we observed that the effect of age on SMN expression significantly depended on the tissue investigated and that the effect of tissue on SMN expression significantly depended on the age investigated (*P* < 0.001). *Post hoc* analyses were performed to determine individual *P*-values for each of the time and tissue comparison we made (Supplementary Material, Table S2). In heart, muscle and kidney, relative SMN expression was <5–10% of control SMN levels, decreasing further over time. In liver, relative SMN expression in SMA was significantly higher than in heart, muscle and kidney. In brain and spinal cord, significantly higher relative levels of SMN were present in SMA mice at P2 that decreased significantly over time to P8. This was in contrast to SMN levels in control animals, where SMN levels initially increased. This implies a particularly high requirement for SMN in nervous tissues and, therefore, a relatively larger effect of SMN depletion on nervous tissues than that on other tissues.

Finally, we were interested to see how reproducible SMN levels were between separate litters of mice. Therefore, we determined SMN expression levels at P8 in a second, independent litter (Fig. 2E) and compared SMN levels to those obtained before (Fig. 2D). Although we observed some modest inter-litter variability, SMN levels between the two litters were comparable and not significantly different in each of the tissues investigated. This confirms that SMN depletion in SMA-like mice is reproducible between litters of the same mouse model.

### *SMN protein expression across tissues in the* Smn^2B/^^−^*model of SMA*

The *Taiwanese* model of SMA is considered a model of severe disease and is characterized by rapid progression leading to death around P9. Requirement for SMN expression, however, is thought to change substantially in the postnatal period up to P20 (25). We therefore wanted to also study SMN protein levels in an intermediate mouse model of SMA: *Smn^2B/^*^*−*^ mice (Fig. 1B, right panel). This model is based on a three-base pair substitution in the exon splicing enhancer region of exon 7 of mouse *Smn* (reffered to as the *Smn^2B^* allele), that leads to *SMN2*-like splicing of the mRNA (26,27). As for *SMN2*, the majority of its protein product is truncated and rapidly degraded (28). *Smn^2B/^*^*−*^ mice are generated by breeding mice that are homozygous for the *Smn^2B^* allele (*Smn^2B/2B^*) with heterozygous *Smn+/−* mice. This leads to experimental litters that consist of ∼50% *Smn^2B/+^* controls and ∼50% *Smn^2B/^*^*−*^ SMA-like mice. At birth, *Smn^2B/^*^*−*^ mice are indistinguishable from littermates, with symptom development starting around P10. After this point, *Smn^2B/^*^*−*^ mice fail to gain weight in the same way as their control littermates. Starting around P15, *Smn^2B/^*^*−*^ mice show extensive pathology at the neuromuscular junction, characterized by denervation, pre-synaptic swelling and poor endplate maturation (29,30). The average survival of *Smn^2B/^*^*−*^ mice varies depending on the genetic background (31). Therefore, for the current experiments, we defined P5 as pre-symptomatic, P10 as early symptomatic and P15 as late symptomatic.

**Figure 3.**
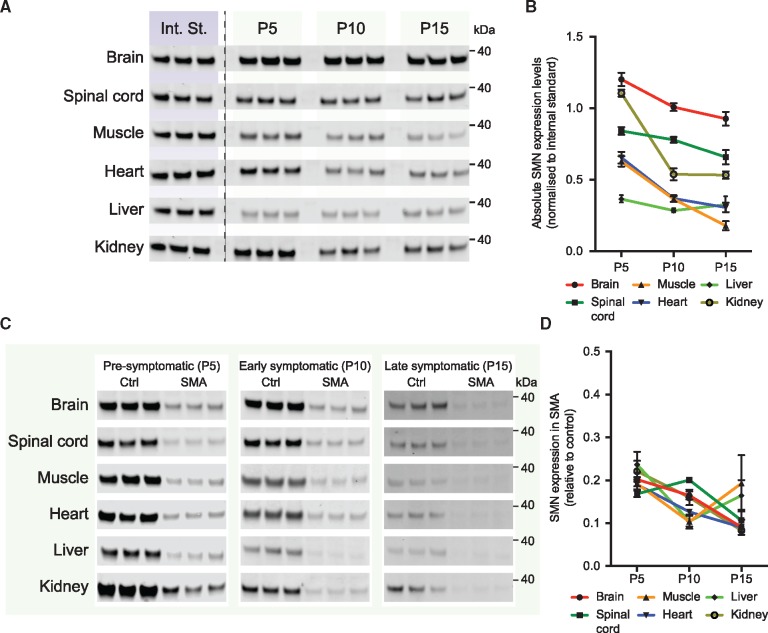
** **Analysis of SMN expression in control mice and Smn^2B/−^ SMA mice. (**A**) SMN expression in control littermates (*Smn+/−; Smn^2B/−^*) of the *Smn^2B/−^* model of SMA was determined using western blot in the indicated tissues. Shown are the Int. St. that was the same for all tissues and loaded in triplicate on each membrane, and SMN expression for each of the tissues at each of the indicated time points (P5, P10 and P15). (**B**) Quantification of SMN expression in all tissues from (A). Expression was first normalized to a fluorescent TPS and then to the Int. St., to allow direct comparison between membranes (*n* = 3 biological replicates for each of the tissues at each of the indicated time points, ± SEM). (**C**) SMN expression is shown for each of the indicated tissues at pre-, early and late-symptomatic ages (P5, P10 and P15) for both control (*Smn+/−; Smn^2B/−^*) and SMA (*Smn−/−; Smn^2B/−^*) mice. (**D**) Quantification of relative SMN depletion in each of the tissues at each of the time points. SMN expression in each sample was first normalized to a fluorescent TPS. SMN expression in SMA littermates is shown relative to control. kDa: 40 kDa molecular weight marker, see Supplementary Material for uncropped western blot membranes.

First, as the time course of the *Smn^2B/^*^*−*^ model is considerably different than that of the *Taiwanese* model, we repeated the experiment in which we determined SMN levels in control littermates (*Smn^2B/+^*, one copy of mouse *Smn* and one copy of the *Smn^2B^* allele) at the disease-relevant time points of P5, P10 and P15. As in the analysis for the *Taiwanese* model, we used brain, spinal cord, muscle (*M. gastrocnemius*), heart, liver and kidney in which we determined SMN protein levels by normalizing to fluorescent TPS and subsequently normalized SMN expression between membranes using an internal protein standard that was analysed in triplicate and three biological replicates for each tissue and each time point (Fig. 3A and B and Supplementary Material, Fig. S3). In the *Smn^2B/^*^*−*^ model of SMA, we found that the effect of age on SMN expression significantly depended upon the tissue investigated and, vice versa, the effect of tissue on SMN expression significantly depended upon the age investigated (*P* < 0.001, Supplementary Material, Table S1). In all tissues except liver SMN levels decreased significantly from P5 to P15, however, SMN levels remained significantly higher in brain and spinal cord than in other tissues at all time points. In other tissues SMN decreased between P5 and P10 and remained more constant from P10 to P15. Liver SMN expression was significantly lower than all other tissues at P5 and remained relatively constant over time.

**Figure 4.**
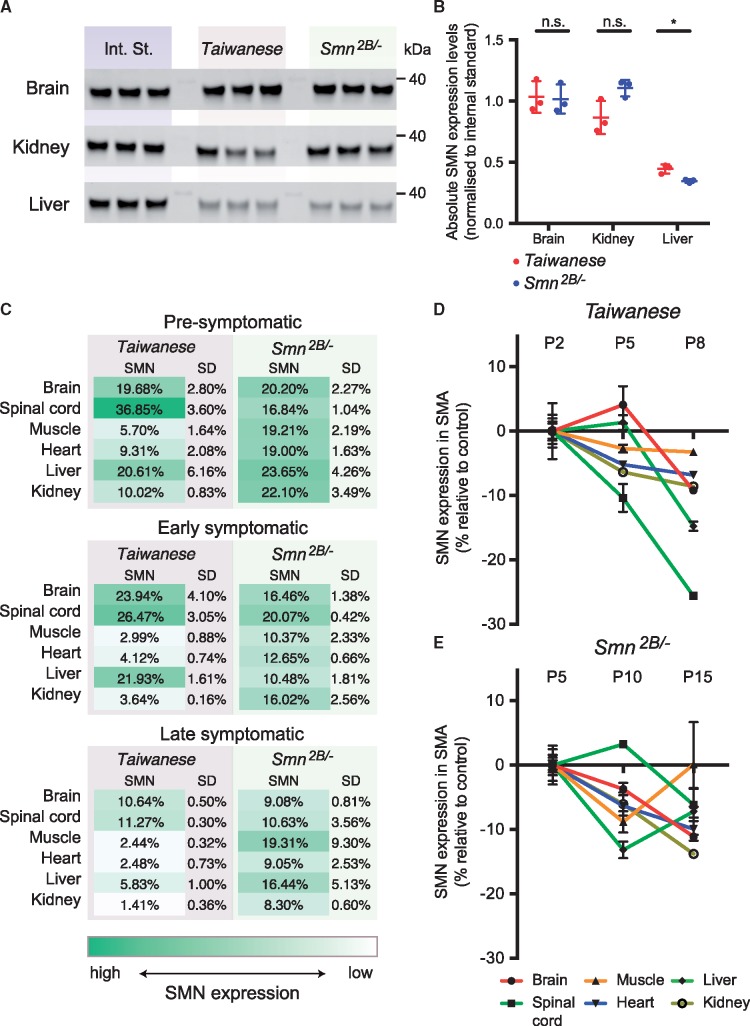
** **Comparison of SMN levels between the Taiwanese and Smn^2B/−^ models of SMA. (**A**) SMN expression was determined by western blot in the indicated tissues at P5 in both mouse models. Shown are the Int. St. that was the same for each tissue and loaded in triplicate on each membrane, and SMN expression in triplicate for each tissue. kDa: 40 kDa molecular weight marker. (**B**) Quantification of SMN expression from the blots in (A), *P < 0.05 (*t*-test). Expression was first normalized to a fluorescent TPS and then to the Int. St., to allow direct comparison between membranes. (**C**) Average of remaining SMN levels (± SD) in both mouse models at each of the investigated time points and tissues. (**D**) Relative depletion in SMN expression over time in *Taiwanese* mice. To allow easier comparison between tissues, P2 was set as 0 and expression values at P5 and P8 are shown relative to P2. (**E**) Relative depletion in SMN expression over time in *Smn^2B/−^* mice. To allow easier comparison between tissues, P5 was set as 0 and expression values at P10 and P15 are shown relative to P5.

After determining control SMN levels, we went on to investigate the extent of SMN depletion at each of the time points in all six tissues of SMA littermates (Fig. 3C and Supplementary Material, Fig. S4). Overall, the relative decrease in SMN expression was similar in all tissues (Fig. 3D and Supplementary Material, Table S2). Relative SMN levels were all ∼20% of control levels at P5 and then decreased significantly over time to ∼10% of control at P15 (for percentages, see also Fig. 4C). Interestingly, however, we did not observe significant differences between tissues at each of the time points. These results indicate that, in the *Smn^2B/^*^*−*^ model of SMA, similar variability of SMN expression occurs between tissues in control mice but that SMN depletion in SMA mice is more comparable between tissues than in the *Taiwanese* model. Interestingly, systemic pathology in the *Taiwanese* model is more pronounced than in the *Smn^2B/^*^*−*^ model (32) and these findings support the idea that this variability is, at least in part, due to variability in SMN expression.

### *Similarities and differences between* Taiwanese *and* Smn^2B/^^*−*^*models of SMA*

We next wanted to investigate possible similarities and differences in SMN expression between the two models in more detail. First, we needed to directly compare SMN expression in the two mouse models by determining SMN levels in brain, kidney and liver in P5 tissue from both models in a single experiment. As before, SMN levels were normalized to TPS and subsequently to an internal standard to account for variability between membranes (Fig. 4A and B and Supplementary Material, Fig. S5). We found that at P5, although protein levels in liver were significantly lower (*P* = 0.013) in *Smn^2B/^*^*−*^ compared with *Taiwanese* mice, SMN levels in both mouse models were highly comparable in each of the tissues investigated.

This finding allowed us to make several observations concerning differences and similarities in SMN protein levels in the *Taiwanese* and *Smn^2B/^*^*−*^ models. First, in control tissues derived from both models, SMN levels in brain and spinal cord were significantly higher than in other tissues. Moreover, SMN expression in kidney was high when compared with other peripheral tissues. Although in the time course relevant to the *Taiwanese* model (P2–P8) SMN expression still increased in control animals, the analysis of the *Smn^2B/^*^*−*^ time course illustrated that around P10, SMN requirements started to decrease and at P15 SMN levels in all tissues were lower than at P5. Surprisingly, when investigating SMN levels in SMA littermates, relative SMN depletion varied significantly between tissues in *Taiwanese* mice but was proportionally decreased in *Smn^2B/^*^*−*^ mice (Fig. 4C). Moreover, when investigating the changes in SMN expression over time in more detail (Fig. 4D and E), we noticed that in *Taiwanese* mice, spinal cord and brain relative SMN expression was comparable at P2 and P5 but then declined sharply between P5 and P8, consistent with disease onset and progression in this model. In *Smn^2B/^*^*−*^ mice, in contrast, SMN levels were comparable across tissues, matching a more modest disease course as observed in this model. The supplementary figures and tables included with this article are provided to allow researchers to make further comparisons between tissues and time points. Because depletion of SMN is central to all pathways and pathologies observed in SMA, these results can provide a starting point for further studies that will help to answer fundamental questions that remain in SMA research.

## Discussion

We here present a comprehensive overview of SMN protein expression variation across different tissues and at different developmental time points in healthy control mice, as well as in two established mouse models of SMA. As SMN levels were determined using robust methodology we were able to make direct and reliable comparisons between a severe and an intermediate SMA model. We found that, in control tissues, SMN levels varied considerably across tissues and time points but were comparable between the two mouse models. However, relative SMN levels in SMA littermates from the same models were substantially different. Moreover, SMN expression levels were found to be consistently higher in CNS tissues, indicating a higher requirement for SMN in neural tissues compared with non-neuronal tissues, comparable with the disease phenotypes observed in SMA patients and mouse models.

SMN protein levels have been reported in several previous studies (summarized in Table 1). Initial studies that used western blot to determine SMN levels in SMA patients often used a variety loading controls and small sample numbers, and the large variation in SMN levels thus obtained is therefore difficult to interpret (28,31,33–36). The number of studies attempting to determine SMN levels over time and in a number of tissues is very limited and again, the use of different techniques [western blot, SMN-electrochemiluminescence (SMN-ECL) and enzyme-linked immunosorbent assay (ELISA)] makes it hard to compare them directly (37–39). Moreover, several studies have focused on determining SMN levels in peripherally accessible tissues and patient cell lines such as blood, fibroblasts and induced-pluripotent stem cell (iPSC)-derived motor neurons, including investigating techniques that allow determining SMN levels in relatively large groups of patients (37,38,40–44). These studies have a primary use for drug and biomarker research and SMN levels in whole blood or blood-derived cells such as lymphoblasts and peripheral blood mononuclear cells (PBMCs) vary quite extensively, seeming to depend partly on the techniques used to determine SMN levels. Our current work overcomes the issues that are present in previous studies by performing standardized western blot quantification methods across tissues, time points and mouse models and thereby allows us to draw more robust conclusions about the expression of SMN protein during normal mouse development and the decrease in SMN protein in SMA littermates. This is an important advance in our understanding of the temporal and tissue requirements of SMN.

Intriguingly, we found that SMN expression in control mice was similar in both models whereas, in contrast, the levels of SMN that remained in SMA littermates were considerably different. It is interesting to speculate what underlies this phenomenon. In a previous study, wild-type mice (with two alleles of mouse *Smn*) were compared with mice that were hemizygous for *Smn* and SMA littermates (37). Here, the authors observed a significant decrease in SMN in SMA littermates but only a small difference between *Smn+/+ *and *Smn+/−* mice. This implies that under control conditions, SMN protein derived from one, wild-type *Smn* allele is sufficient for most cell types and therefore the presence of two copies of *SMN2* (*Taiwanese* model) or a mouse-derived mutant *Smn* allele (*Smn^2B/^*^*−*^ model) does not further significantly influence SMN levels. However, as in SMA mice *all* full-length SMN is derived exclusively from the *SMN2* and *Smn^2B/^*^*−*^ alleles, SMN protein levels in SMA conditions highlight the differential processing and translation of the *SMN* mRNAs that are derived from these alleles in different tissues. Further studies are now warranted to identify the cellular mechanisms that underlie these differences, as they could provide additional therapeutic targets for increasing SMN levels.

Although motor neurons are the primary pathological target in SMA, other tissues have also shown to be affected, both in patients and in animal models (11,45). This study highlights further how substantial levels of SMN are present systemically. When investigating defects in specific tissues in SMA, the dataset presented in this article provides a resource for interpreting the results in the context of overall SMN expression. Moreover, the results are in line with a threshold model for SMN requirement, in which CNS tissues are more sensitive to SMN depletion than other tissues, as has been suggested previously (46). However, our findings indicate that significant variability can be present between tissues and mouse models, and that considerable changes in expression occur over time, which should be taken into account when interpreting results obtained from individual tissues at specific time points. Future research may be able to add further detail to these findings by investigating the effect of SMN expression on specific cell types, such as vulnerable and resistant populations of spinal motor neurons (29,47,48).

In line with the large variety of systemic defects that is observed in SMA, many cellular pathways have been identified to be affected by SMN (14,15,49). Although these findings provide important novel insights into the pathogenesis of SMA, to really advance our understanding of SMA it is becoming increasingly necessary to search for and identify central regulators of the SMA disease process. Depletion of SMN in different tissues is central to all these pathways and therefore a better understanding of SMN expression over time as presented in this article can provide a starting point for further studies. These studies will help in answering vital questions about SMA pathogenesis that will in turn assist in further optimization and development of current and future therapies.

## Materials and Methods

### Mouse models and tissue collection

All animal work was performed in concordance with institutional and Home Office regulations (PPL 60/4569). All mice were housed at the University of Edinburgh under standard specific pathogen-free conditions. The *Taiwanese* model of SMA was originally obtained from Jackson Laboratories (strain 005058) and maintained as described previously (22,23). Mice carrying the *Smn^2B^* allele backcrossed onto a C57BL6 background were originally obtained from Dr Rashmi Kothary, University of Ottawa, Canada and bred with *Smn+/−* mice obtained from Jackson Laboratories (strain 10921). Brain, spinal cord, muscle (*M. gastrocnemius*), heart, liver and kidney were quickly dissected following humane euthanasia, immediately snap-frozen on dry ice and stored at −80°C until required for western blot analysis. In SMA littermates from the *Taiwanese* model full-length SMN is exclusively derived from the human *SMN2* transgene whereas, in the *Smn^2B/^*^*−*^ model, full-length protein is derived from a mouse allele. For consistency, we refer to SMN rather than Smn throughout the article.

### Quantitative western blotting

For western blot analysis, tissues were thawed on ice and washed three times in ice cold PBS. Tissues were homogenized on ice using a pestle homogenizer in radioimmunoprecipitation assay buffer (RIPA buffer) (ThermoFisher Scientific) with protease inhibitor (Halt Protease Inhibitor Cocktail, ThermoFisher Scientific). Sample homogenates were incubated on ice for 10 min before being centrifuged for 10 min at 4°C at >13 000*g*. Cleared tissue lysates were moved to a new tube and protein concentrations were determined using the bicinchoninic acid method (BCA method) (ThermoFisher Scientific). Protein concentration was normalized for all tissues at 2.5 µg/µl in MilliQ water with 4x SDS sample buffer (ThermoFisher Scientific) containing β-mercaptoethanol (Sigma-Aldrich) as a reducing agent. For all samples and tissue types, 20 µg of protein was loaded onto a 4–12% Bis-Tris gradient gel (ThermoFisher Scientific), and samples were size separated by running the gel for 10 min at 80V followed by 150V for 60 min. Proteins were subsequently transferred to a polyvinylidene difluoride (PDVF) membrane using the iBlot2 semi-dry blotting system (ThermoFisher Scientific). Immediately after transfer, PDVF membranes were incubated in Revert Total Protein Stain (Li-Cor) for 5 min at room temperature (RT), washed and blocked in Odyssey PBS blocking buffer (Li-Cor) for 30 min at RT. All membranes were incubated in freshly made up SMN-antibody solution (mouse-anti-SMN, BD Bioscience 610646, 1:1000 in Odyssey blocking buffer) and incubated overnight at 4°C. After primary antibody incubation, membranes were washed 3 × 10 min in 1× PBS at RT and incubated in donkey-anti-mouse IRDye 800 (Li-Cor) secondary antibody at 1:5000 in Odyssey blocking buffer for 1 h at RT. Membranes were washed 3 × 30 min in 1× PBS at RT and dried followed by image acquisition on an Odyssey CLX laser-based scanning system (Li-Cor).

### Quantification and normalization of SMN levels across tissues

For all analyses, TPS and anti-SMN intensity were first determined using ImageStudio (Li-Cor) and SMN levels were normalized to TPS signal intensity to control for protein loading variation. To determine the relative decrease of SMN expression in SMA, average SMN expression for three control mice was calculated and defined as 1. SMN expression in SMA mice was subsequently calculated by dividing the normalized expression in SMA samples by the average normalized expression in controls. This returns a normalized, relative expression value for each of the SMA samples analysed. To be able to directly compare SMN expression across tissues and over time in control tissues, all time points for each tissue were analysed on one western blot membrane, in addition to an internal standard that was the same across all membranes and tissues, and analysed concurrently with each of the time points. The internal standard consisted of a mixture of brain protein lysates, because SMN levels in brain are high and it is possible to obtain large quantities of protein from brain, thereby allowing us to make a large quantity of our internal standard that could be used consistently across all membranes. The internal standard was loaded in triplicate on all membranes and allowed us to correct for technical variation that could have occurred in handling and processing the individual western blotting membranes. After normalizing SMN expression levels using TPS intensity for all samples, including the internal standard, the average intensity value of the internal standard was calculated and defined as 1 on each of the membranes. The normalized intensity values for SMN where subsequently divided by the average value of the internal standard on each of the membranes, thus allowing to compare SMN levels across tissues. These values were used in the further statistical analyses as described later.

### Statistical analysis

To statistically determine the effects of tissue and age on SMN expression, we modelled the expression using a mixed-effects model, implemented in lmer (lme4 package in R) (50). The model specified tissue type, age and the interaction between tissue and age as fixed effects, and mouseID as a random effect to account for non-independence of multiple measurements from individual mice. As expression data for *Taiwanese* mice did not meet the assumptions of homogeneity of variance and normality of the analysis, we square-root transformed the expression data to better meet the assumptions of model residuals to the data. To test for a significant interaction, we fitted the model a second time, excluding the interaction and then used parametric bootstrapping (using PBmodcomp from the pbkrtest package in R) to compare the models. To determine the differences between individual time points and tissues, we compared SMN expression between all combinations of age (i.e. 2 versus 5, 2 versus 8, 5 versus 8 for *Taiwanese* and 5 versus 10, 5 versus 15, 10 versus 15 for *Smn2^B/^*^*−*^), and all combinations of tissues (brain versus spinal cord, muscle, heart, liver, kidney, spinal cord versus muscle, heart, liver, kidney, etc.) performed separately for each tissue (with the emmeans package in R using Tukey *post hoc* analysis).

## Supplementary Material

Supplementary Material is available at *HMG* online.

*Conflict of Interest statement*. None declared.

## Funding

This work was funded by grants from the Wellcome Trust (106098/Z/14/Z), the SMA Trust (UK SMA Research Consortium), the Euan MacDonald Centre for Motor Neurone Disease Research and SMA Europe. Funding to pay the Open Access publication charges for this article was provided by the Charity Open Access Fund (Wellcome Trust).

## Supplementary Material

Supplementary Data
