# Supplementary material for: Temporal and tissue-specific variability of SMN protein levels in mouse models of spinal muscular atrophy
Source: Hum Mol Genet. 2018 May 22;27(16):2851–62. doi: 10.1093/hmg/ddy195 (PMC6077828; doi:10.1093/hmg/ddy195)
Supplement: Supplementary Data [file ddy195_supp.pdf]

Supplementary information for:

Temporal and tissue-specific variability of SMN protein levels in mouse models of spinal muscular atrophy

Ewout JN Groen, Elena Perenthaler, Natalie Courtney, Crispin Y Jordan,
Hannah K Shorrock, Dinja van der Hoorn, Yu-Ting Huang, Lyndsay M Murray,
Gabriella Viero, Thomas H Gillingwater

Contents

- Legends to supplementary figures and tables
- Supplementary figures 1-5
- Supplementary tables 1-2

Legends to supplementary figures

Supplementary figures 1 and 3.

Uncropped SMN Western blots (top panels) and total protein stain (bottom panels) for the indicated tissues for the *Taiwanese* (Figure S1) and *Smn*$^{2B/-}$ (Figure S3) mouse models of SMA. The investigated developmental time points are indicated above the Western blot panels. The internal standard (int. st.) is the same on all membranes and was loaded as a triplicate on the left of all membranes. The 40 kDa marker from the protein standard that was loaded between each of the time points and the internal standard is indicated for all membranes (SMN runs at ~35-36 kDa). IB: immunoblot, TPS: total protein stain, P(2, 5, 8; 5, 10, 15): postnatal day.

Supplementary figures 2 and 4.

Uncropped SMN Western blots (top panels) and total protein stain (bottom panels) for the indicated tissues at the indicated time points for the *Taiwanese* (Figure S2) and *Smn*$^{2B/-}$ (Figure S4) mouse models of SMA. For each tissue, 3 control (Ctrl) and 3 SMA littermates were analysed. The 40 kDa marker from the protein standard that was loaded between each of the time points and the internal standard is indicated for all membranes (SMN runs at ~35-36 kDa). IB: immunoblot, TPS: total protein stain.

Supplementary figure 5.

Uncropped SMN Western blots (top panels) and total protein stain (bottom panels) for the indicated tissues. The investigated developmental mouse models are indicated above the Western blot panels. The internal standard (int. st.) is the same on all membranes and was loaded as a triplicate on the left of all membranes. The 40 kDa marker from the protein standard that was loaded between each of the time points and the internal standard is indicated for all membranes (SMN runs at ~35-36 kDa). Taiw.: *Taiwanese*, IB: immunoblot, TPS: total protein stain, P(2, 5, 8; 5, 10, 15): postnatal day.

Supplementary figure 1.
Uncropped SMN Western blots (Taiwanese) and fluorescent total protein stain (TPS)
Corresponding to Figure 2A and 2B

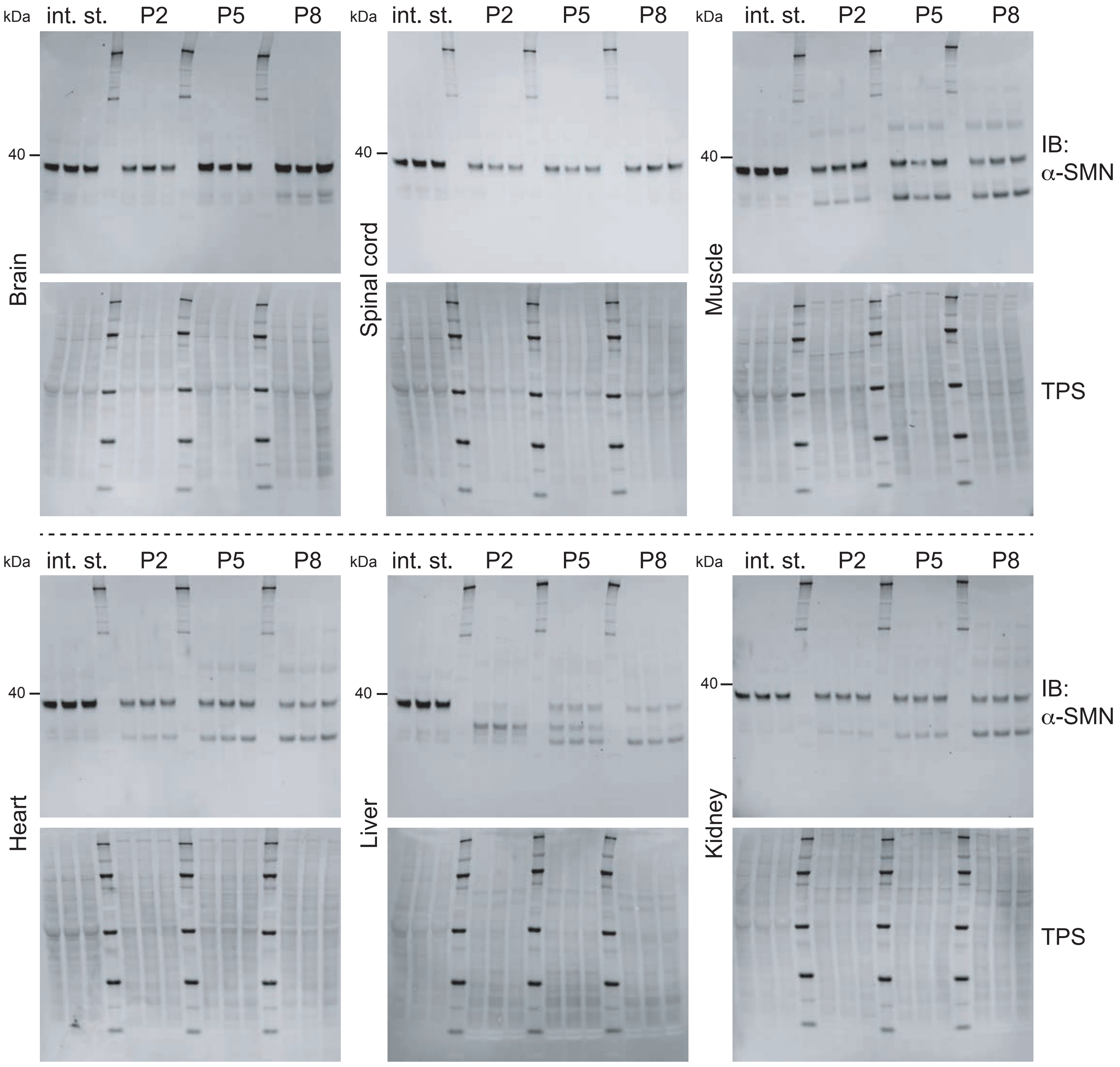

Supplementary figure 2.
Uncropped SMN Western blots (Taiwanese) and fluorescent total protein stain (TPS)
Corresponding to Figure 2C and 2D

Postnatal day 2 (P2)

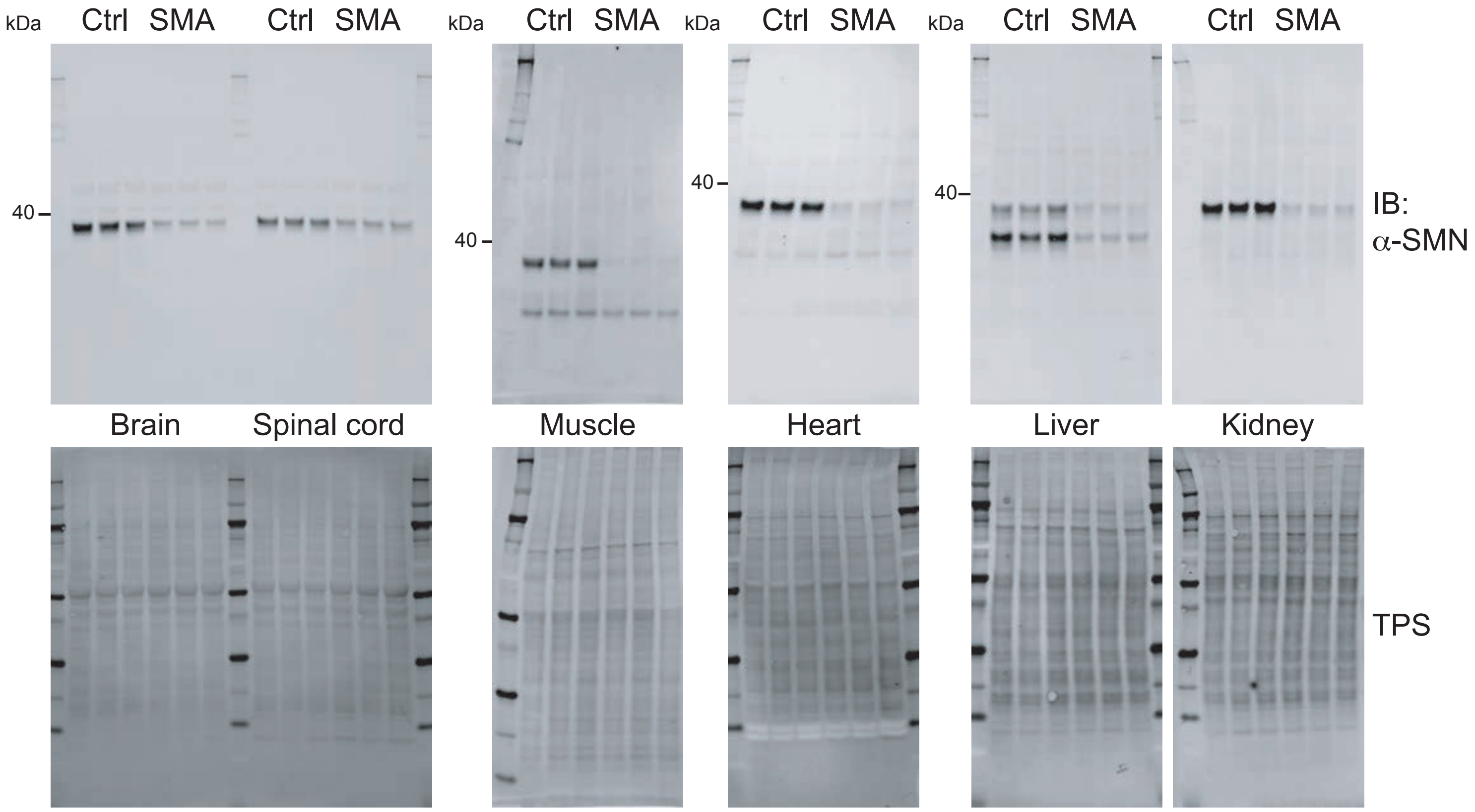

Postnatal day 5 (P5)

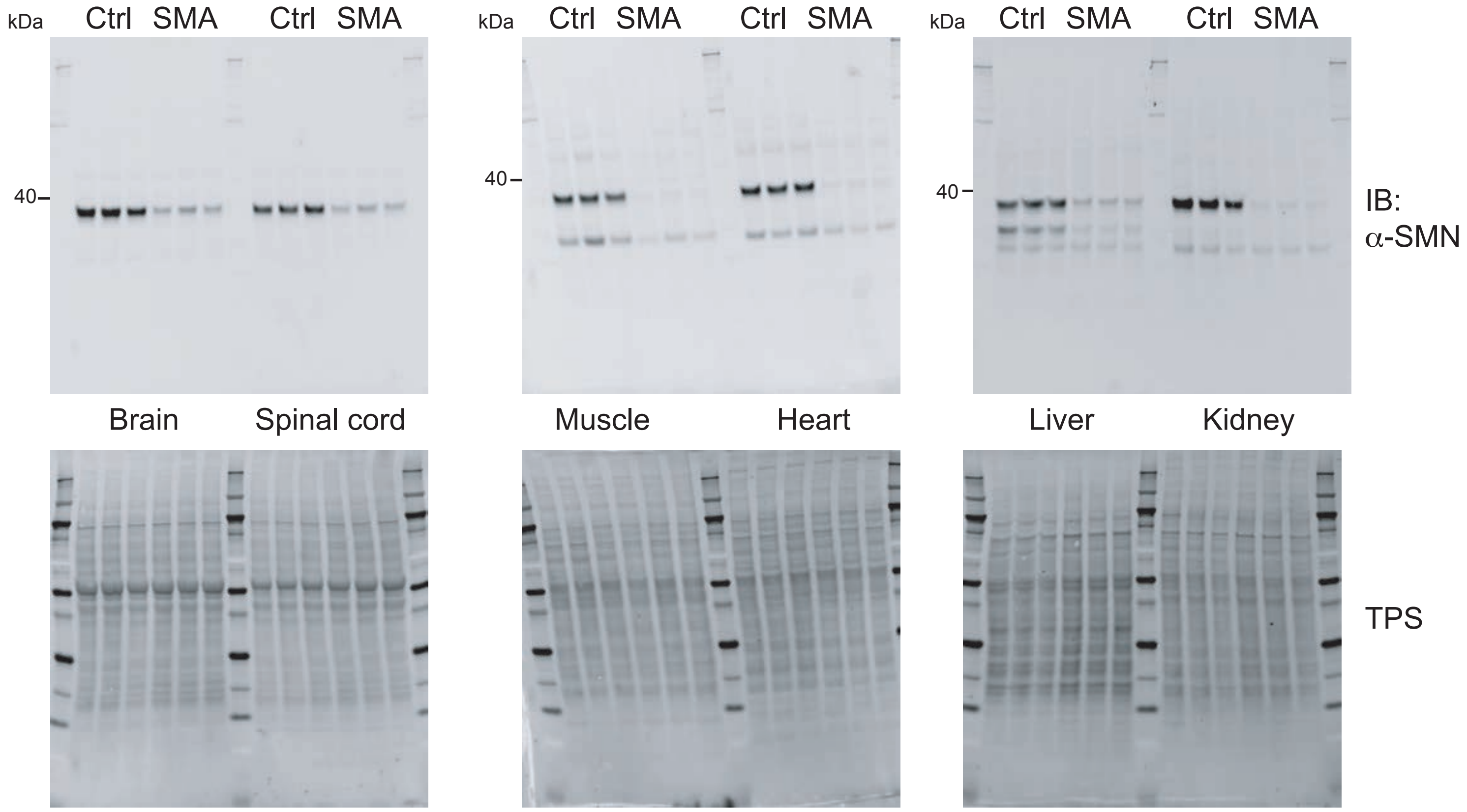

Supplementary figure 2 (continued)
Uncropped SMN Western blots (Taiwanese) and fluorescent total protein stain (TPS)
Corresponding to Figure 2C and 2D

Postnatal day 8 (P8)

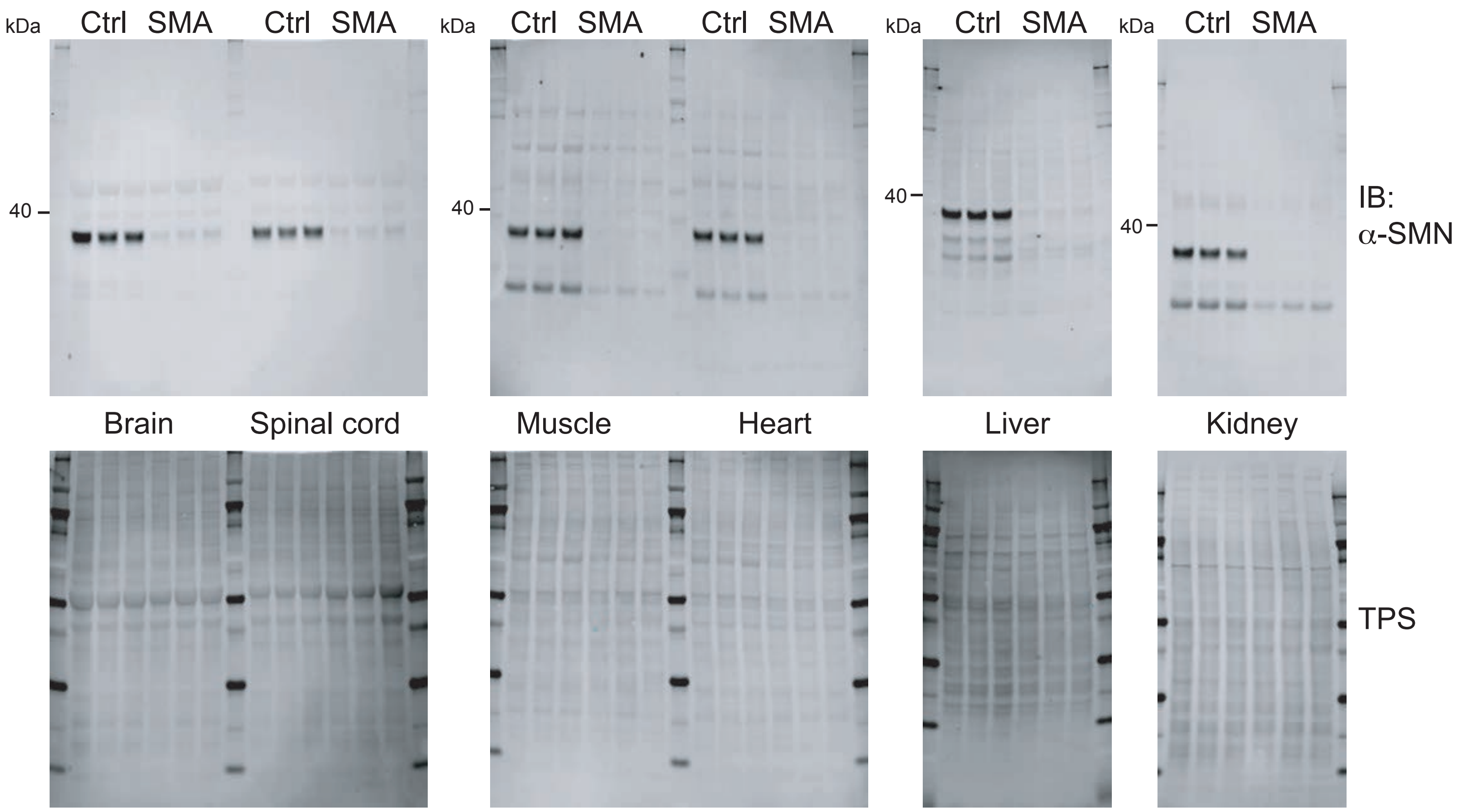

Supplementary figure 3.

Uncropped SMN Western blots (Smn2B/-) and fluorescent total protein stain (TPS)

Corresponding to Figure 3A and 3B

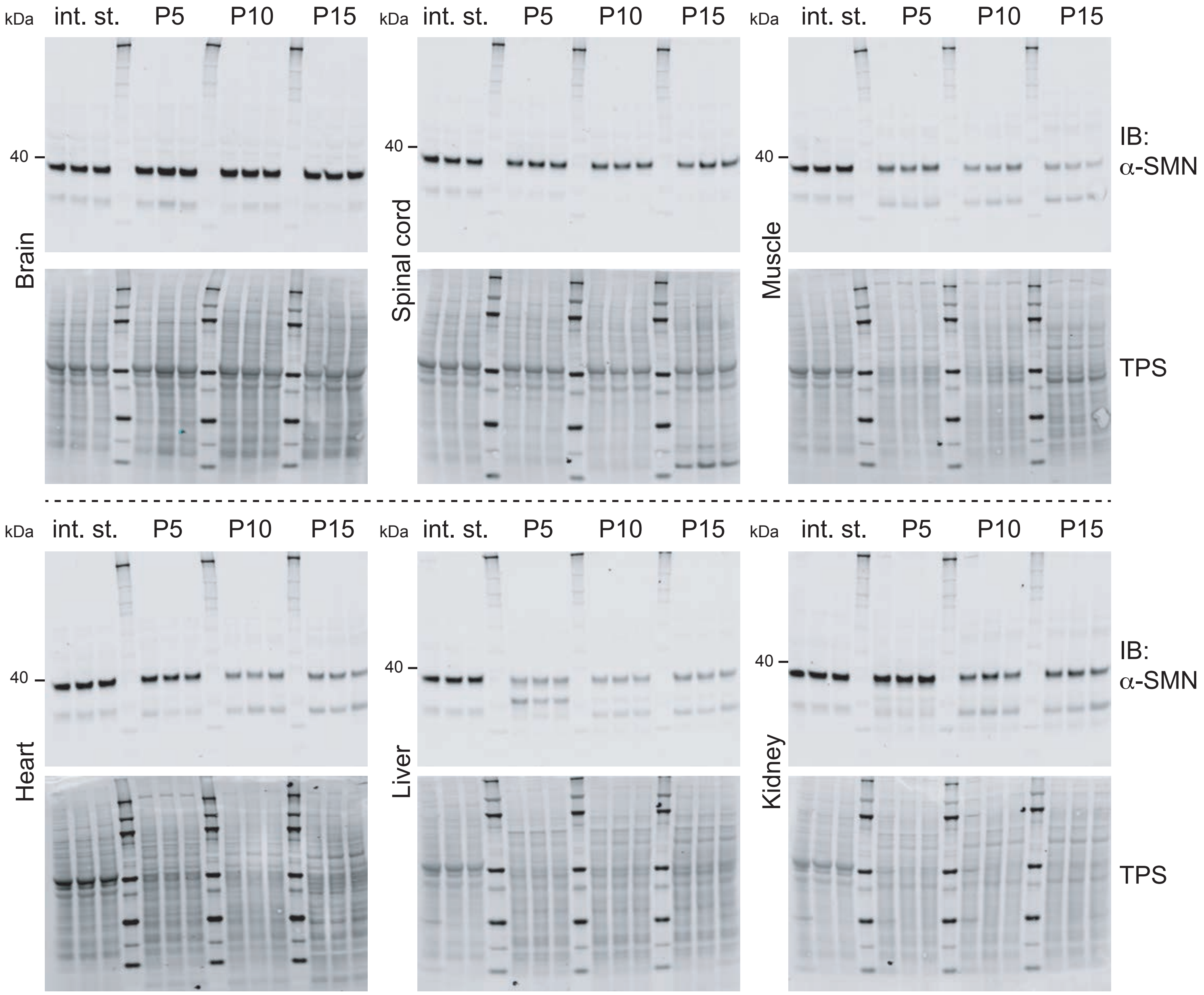

Supplementary figure 4.
Uncropped SMN Western blots (Smn2B/-) and fluorescent total protein stain (TPS)
Corresponding to Figure 3C and 3D

Postnatal day 5 (P5)

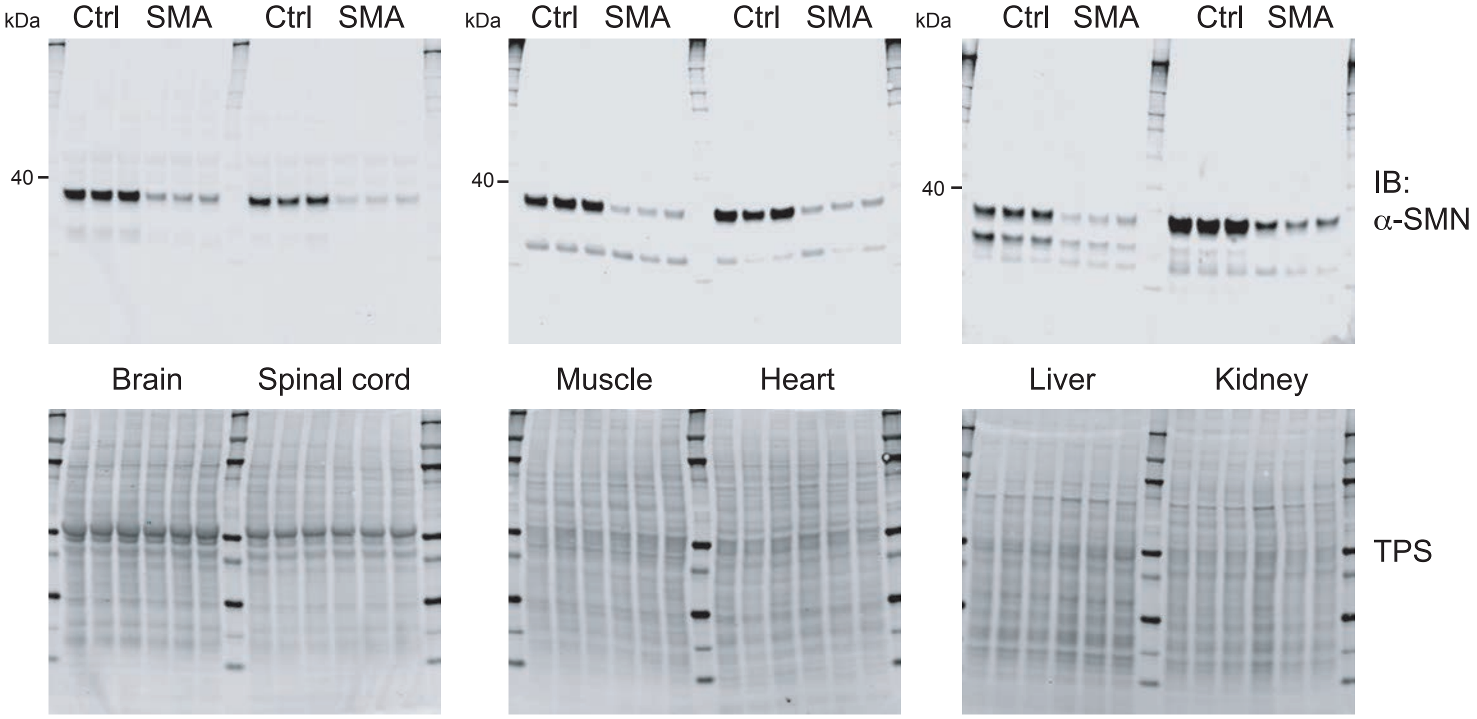

Postnatal day 10 (P10)

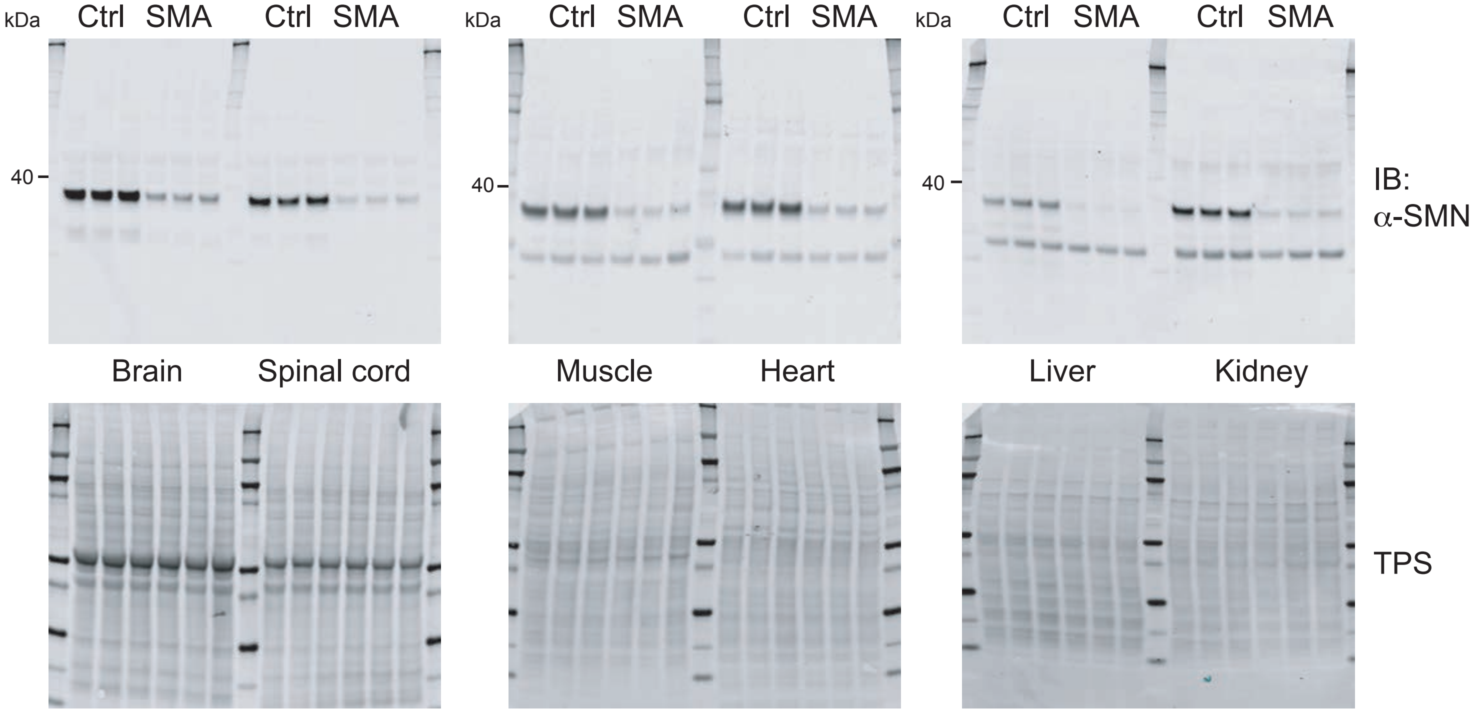

Supplementary figure 4 (continued)
Uncropped SMN Western blots (Smn2B/-) and fluorescent total protein stain (TPS)
Corresponding to Figure 3C and 3D

Postnatal day 15 (P15)

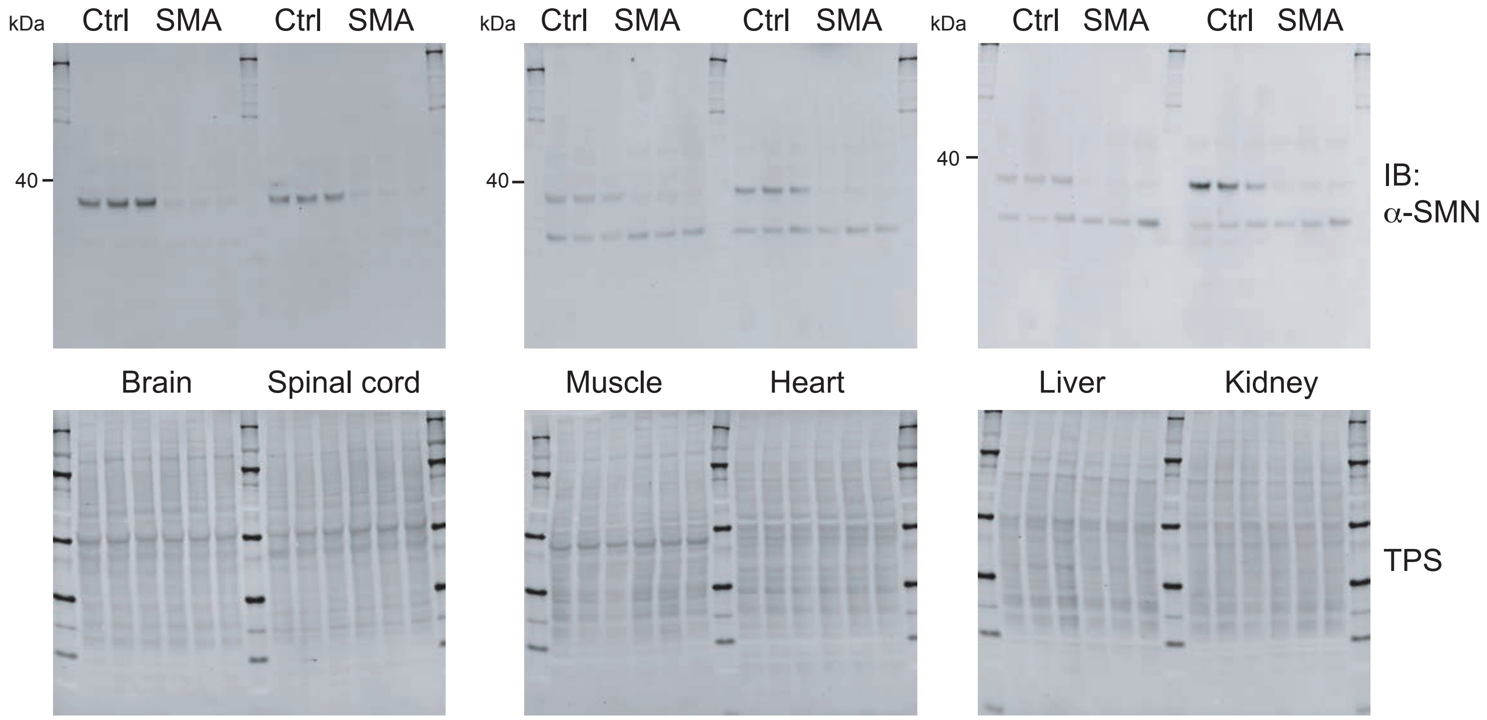

Supplementary figure 5.
Uncropped SMN Western blots and fluorescent total protein stain (TPS)
Corresponding to Figure 4A

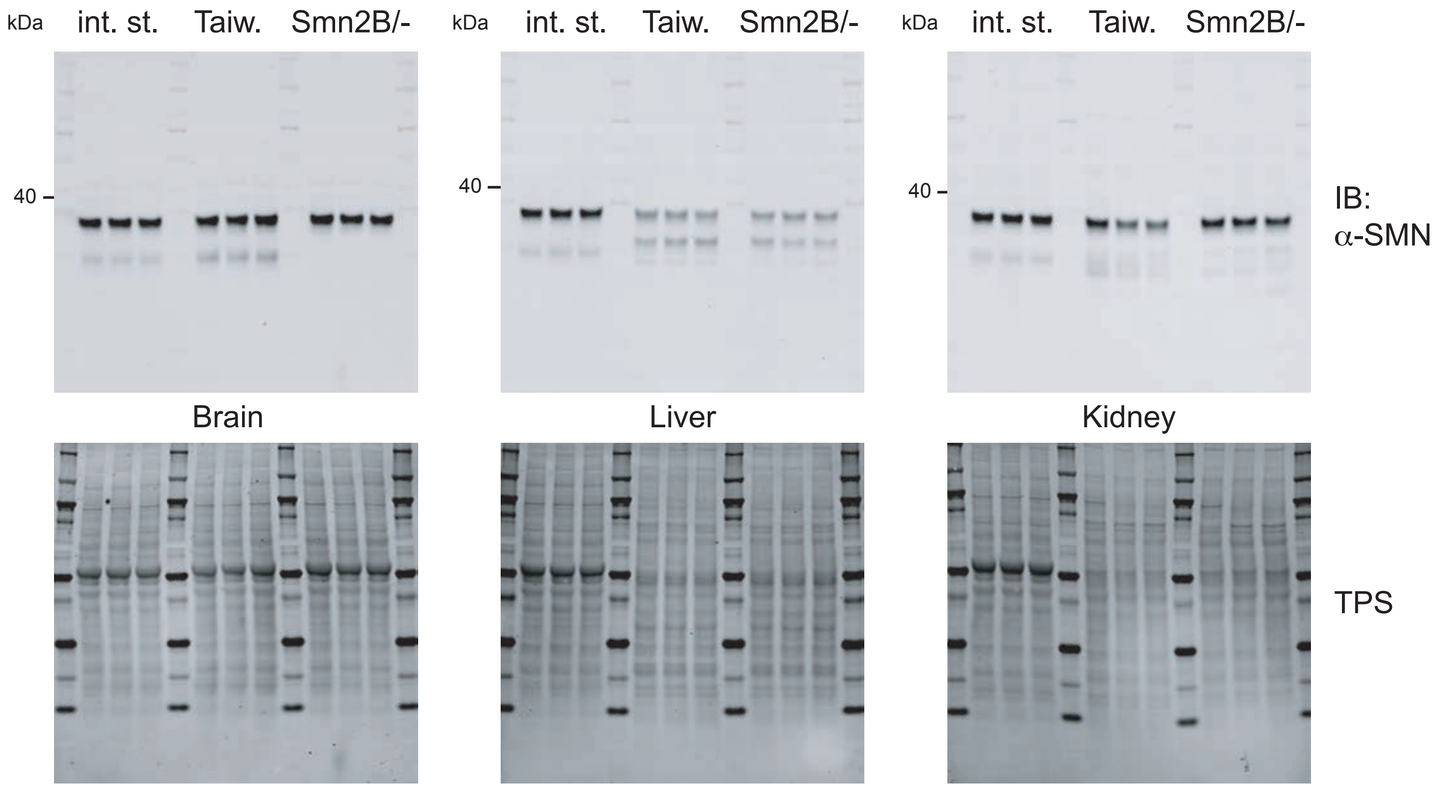

Supplementary table 1. P-values for time point and tissue comparison for control SMN levels (corresponding to Figure 2A-B and 3A-B)

a. *Taiwanese* - time points comparison

Brain	
P2 vs P8	**<.0001**
P2 vs P5	**0.0013**
P5 vs P8	0.3042

Heart	
P2 vs P8	0.181
P2 vs P5	0.9982
P5 vs P8	0.1633

Spinal cord	
P2 vs P8	**0.0443**
P2 vs P5	0.9905
P5 vs P8	0.0594

Liver	
P2 vs P8	**0.0016**
P2 vs P5	**<.0001**
P5 vs P8	0.4196

Muscle	
P2 vs P8	**0.0025**
P2 vs P5	0.1043
P5 vs P8	0.2888

Kidney	
P2 vs P8	**0.0304**
P2 vs P5	0.3526
P5 vs P8	0.4244

b. *Smn*$^{2B/-}$- time points comparison

Brain	
P5 vs P15	**<.0001**
P5 vs P10	**0.0005**
P10 vs P15	0.1902

Heart	
P5 vs P15	**<.0001**
P5 vs P10	**<.0001**
P10 vs P15	0.3746

Spinal cord	
P5 vs P15	**0.0008**
P5 vs P10	**0.0315**
P10 vs P15	0.368

Liver	
P5 vs P15	0.6773
P5 vs P10	0.1974
P10 vs P15	0.6349

Muscle	
P5 vs P15	**<.0001**
P5 vs P10	**0.0007**
P10 vs P15	**<.0001**

Kidney	
P5 vs P15	**<.0001**
P5 vs P10	**<.0001**
P10 vs P15	0.9935

c. *Taiwanese* - tissues comparison

**P2**	Brain	SC	Muscle	Heart	Liver	Kidney
Brain						
SC	0.1415					
Muscle	0.2375	0.9997				
Heart	**0.0029**	0.5877	0.4169			
Liver	**<.0001**	**<.0001**	**<.0001**	**<.0001**		
Kidney	1.0000	0.1209	0.2066	**0.0024**	**<.0001**	

**P5**	Brain	SC	Muscle	Heart	Liver	Kidney
Brain						
SC	**<.0001**					
Muscle	**<.0001**	0.3637				
Heart	**<.0001**	0.5399	0.9996			
Liver	**<.0001**	**<.0001**	**0.0006**	**0.0003**		
Kidney	**0.0001**	0.8919	**0.0471**	0.0912	**<.0001**	

**P8**	Brain	SC	Muscle	Heart	Liver	Kidney
Brain						
SC	**0.0001**					
Muscle	**<.0001**	**<.0001**				
Heart	**<.0001**	**<.0001**	1.0000			
Liver	**<.0001**	**<.0001**	**0.0012**	**0.0014**		
Kidney	**<.0001**	0.1146	**0.0248**	**0.0223**	**<.0001**	

d. *Smn*$^{2B/-}$- tissues comparison

**P5**	Brain	SC	Muscle	Heart	Liver	Kidney
Brain						
SC	**<.0001**					
Muscle	**<.0001**	**0.0008**				
Heart	**<.0001**	**0.0036**	0.9925			
Liver	**<.0001**	**<.0001**	**<.0001**	**<.0001**		
Kidney	0.3104	**<.0001**	**<.0001**	**<.0001**	**<.0001**	

**P10**	Brain	SC	Muscle	Heart	Liver	Kidney
Brain						
SC	**0.0003**					
Muscle	**<.0001**	**<.0001**				
Heart	**<.0001**	**<.0001**	1.0000			
Liver	**<.0001**	**<.0001**	0.5021	0.4548		
Kidney	**<.0001**	**0.0001**	**0.0080**	**0.0097**	**0.0001**	

**P15**	Brain	SC	Muscle	Heart	Liver	Kidney
Brain						
SC	**<.0001**					
Muscle	**<.0001**	**<.0001**				
Heart	**<.0001**	**<.0001**	0.0868			
Liver	**<.0001**	**<.0001**	**0.0325**	0.9978		
Kidney	**<.0001**	0.0971	**<.0001**	**0.0003**	**0.0011**	

Supplementary table 2. P-values for time point comparisons for remaining SMN levels in SMA mice (corresponding to Figure 2C-D and 3C-D)

a. *Taiwanese* - time points comparison

Brain		*Heart*	
P2 vs P8	**0.0014**	P2 vs P8	**0.0171**
P2 vs P5	0.6937	P2 vs P5	0.0798
P5 vs P8	**0.0002**	P5 vs P8	0.756

Spinal cord		*Liver*	
P2 vs P8	**<.0001**	P2 vs P8	**<.0001**
P2 vs P5	**0.0004**	P2 vs P5	0.8315
P5 vs P8	**<.0001**	P5 vs P8	**<.0001**

Muscle		*Kidney*	
P2 vs P8	**0.0004**	P2 vs P8	**0.0027**
P2 vs P5	**0.0008**	P2 vs P5	**0.0268**
P5 vs P8	0.969	P5 vs P8	0.5971

b. *Smn*$^{2B/-}$- time points comparison

Brain		*Heart*	
P5 vs P15	**0.0004**	P5 vs P15	**0.0015**
P5 vs P10	0.3224	P5 vs P10	**0.0486**
P10 vs P15	**0.0193**	P10 vs P15	0.3486

Spinal cord		*Liver*	
P5 vs P15	0.0548	P5 vs P15	**0.0226**
P5 vs P10	0.4248	P5 vs P10	**<0.0001**
P10 vs P15	**0.0025**	P10 vs P15	0.0666

Muscle		*Kidney*	
P5 vs P15	N/A	P5 vs P15	**<0.0001**
P5 vs P10	N/A	P5 vs P10	0.0607
P10 vs P15	N/A	P10 vs P15	**0.014**

c. *Taiwanese* - tissues comparison

**P2**	Brain	SC	Muscle	Heart	Liver	Kidney
Brain						
SC	**<.0001**					
Muscle	**0.0093**	**<.0001**				
Heart	**0.0001**	**<.0001**	0.4750			
Liver	0.9987	**<.0001**	**0.0033**	**<.0001**		
Kidney	**0.0002**	**<.0001**	0.7046	0.9990	**0.0001**	

**P5**	Brain	SC	Muscle	Heart	Liver	Kidney
Brain						
SC	0.1589					
Muscle	**<.0001**	**<.0001**				
Heart	**<.0001**	**<.0001**	0.9906			
Liver	1.0000	**<.0001**	0.1931	**<.0001**		
Kidney	**<.0001**	**<.0001**	0.9993	0.9999	**<.0001**	

**P8**	Brain	SC	Muscle	Heart	Liver	Kidney
Brain						
SC	0.9994					
Muscle	**0.0020**	**0.0008**				
Heart	**0.0022**	**0.0009**	1.0000			
Liver	0.0567	**0.0290**	0.9628	0.9664		
Kidney	**0.0005**	**0.0002**	0.9938	0.9926	0.7872	

d. *Smn*$^{2B/-}$ - tissues comparison

**P5**	Brain	SC	Heart	Liver	Kidney
Brain					
SC	0.6440				
Heart	0.9871	0.8987			
Liver	0.6248	0.0685	0.3394		
Kidney	0.9346	0.2285	0.7096	0.9675	

**P10**	Brain	SC	Heart	Liver	Kidney
Brain					
SC	0.5836				
Heart	0.5339	**0.0404**			
Liver	0.1345	**0.0051**	0.8963		
Kidney	0.9997	0.4741	0.6448	0.1874	

**P15**	Brain	SC	Heart	Liver	Kidney
Brain					
SC	0.9676				
Heart	1.0000	0.9650			
Liver	**0.0426**	0.1541	**0.0413**		
Kidney	0.9976	0.8717	0.9979	**0.0208**	
